# Advances in endobariatrics: past, present, and future

**DOI:** 10.1093/gastro/goad043

**Published:** 2023-07-21

**Authors:** Abhishek Shenoy, Allison R Schulman

**Affiliations:** Division of Gastroenterology and Hepatology, University of Michigan, Michigan Medicine, Ann Arbor, MI, USA; Division of Gastroenterology and Hepatology, University of Michigan, Michigan Medicine, Ann Arbor, MI, USA; Department of Surgery, University of Michigan, Michigan Medicine, Ann Arbor, MI, USA

**Keywords:** metabolic devices, endoscopic sleeve gastroplasty, obesity, endobariatrics, bariatric therapy

## Abstract

The obesity epidemic in the USA and worldwide is well documented and continues to grow. Endoscopic metabolic and bariatric therapies may offer a less invasive approach than surgical intervention. This article will review advances in endobariatrics over the last several decades, addressing the past and current state of bariatric and metabolic endoscopy. Food and Drug Administration-cleared devices and interventions currently under investigation are described including gastric devices, gastric remodeling procedures, small-bowel devices, duodenal ablation, as well as procedures to address weight regain after bariatric surgery. Future studies evaluating gastric and duodenal combination therapy, adjunctive pharmacotherapy, as well as individualized precision-health algorithms are underway.

## Introduction

The obesity epidemic in the USA and worldwide continues to grow. The prevalence of obesity has increased in every country in the world since 1975 [1]. Obesity may lead to co-morbidities including but not limited to metabolic syndrome, insulin resistance and type 2 diabetes mellitus (T2DM), and non-alcoholic fatty liver disease [2]. It is predicted that, by 2030, the USA will have an obesity rate of 48.9%, meaning that almost one in two adults will be classified as obese, and ≤24.2%, or nearly one in four adults, will have severe obesity [3].

As obesity rates rise, there remains a significant impact on the healthcare system. Its impact is not only on resource utilization from obesity-related chronic disease states, but also on financial burden to each individual patient and the healthcare system as a unit. It has been shown that associated healthcare costs are proportionally increased with each increased unit in body mass index (BMI). On average, adults with obesity were associated with $1,861 of excess annual medical costs per person accounting for $172.74 billion of expenditures annually [4]. A majority of these costs are indirect due to obesity-related co-morbidities; nevertheless, curbing the obesity epidemic may have a significant impact on improving the system overall.

Effective approaches that are minimally invasive include lifestyle interventions such as diet and exercise; however, even when maximum patient effort is achieved, these interventions on average only lead to 3%–5% of total body weight loss [5, 6]. Pharmacologic therapy has become increasingly effective for weight loss, but these medications can be poorly tolerated, expensive, challenging to access, and with limited long-term data.

The most effective treatment modality for patients with obesity is bariatric surgery. Unfortunately, it is well known that only 1%–2% of eligible patients undergo surgical management for obesity, despite its effectiveness with respect to both obesity-related co-morbidities and weight loss [7].

Endoscopic metabolic and bariatric therapies may offer a less invasive approach than surgical intervention. Endoscopic metabolic and bariatric therapies includes gastric devices and techniques that are Food and Drug Administration (FDA)-cleared including space-occupying devices and gastric remodeling procedures, and small-bowel interventions that are currently under investigation and aimed at treating obesity-related co-morbidities. These procedures may be an option for patients who are reluctant to undergo surgery or who are poor surgical candidates. The field of endobariatrics may help to bridge this gap significantly. Growth in providers, research and development, and novel devices over the last two decades have helped evolve the field into what it is today.

In this paper, we will review advances in endobariatrics over the last several decades, addressing the past, present, and future of bariatric and metabolic surgery. We will review FDA-cleared devices and interventions currently under investigation.

## Gastric devices

### Space-occupying devices

Numerous studies have demonstrated the safety and efficacy of space-occupying devices as an effective method to induce weight loss. These devices are known to work through volume restriction and early satiety, but also involve delayed gastric emptying and a complex interplay of hormonal changes that promotes weight loss.

#### Intragastric balloons

Intragastric balloons (IGBs) are space-occupying devices temporarily placed within the lumen of the stomach. Though IGBs were manufactured and launched decades ago, only recently have these devices gained popularity in the USA and internationally as a plausible option for patients in search of a weight-loss strategy. In 1984, the first IGB known as the Garren-Edwards bubble was manufactured and was FDA-approved for temporary use as a weight-loss device [8]. Approval was subsequently withdrawn in 1992 due to several complications and poor weight-loss efficacy. Much progress has been made over the last several decades with four FDA-cleared IGBs and several more in development. Recent American Gastroenterological Association guidelines had a conditional recommendation for the use of intragastric balloon therapy over lifestyle modification alone in patients seeking weight loss who had failed conventional therapy.

In this section, we will focus on the ORBERA™ Intragastric Balloon System (OIBS; Apollo Endosurgery, Inc., Austin, TX, USA), Spatz3™ balloon (Spatz Medical, Fort Lauderdale, FL, USA), ReShape^®^ Integrated Dual Balloon System (ReShape Medical, Inc., San Clemente, CA, USA), Obalon (Obalon^®^ Therapeutics, Inc., Carlsbad, CA, USA), and Elipse (Allurion Technologies, Natick, MA, USA), though not all are commercially available. We will discuss each balloon in detail below.

**Figure 1.**
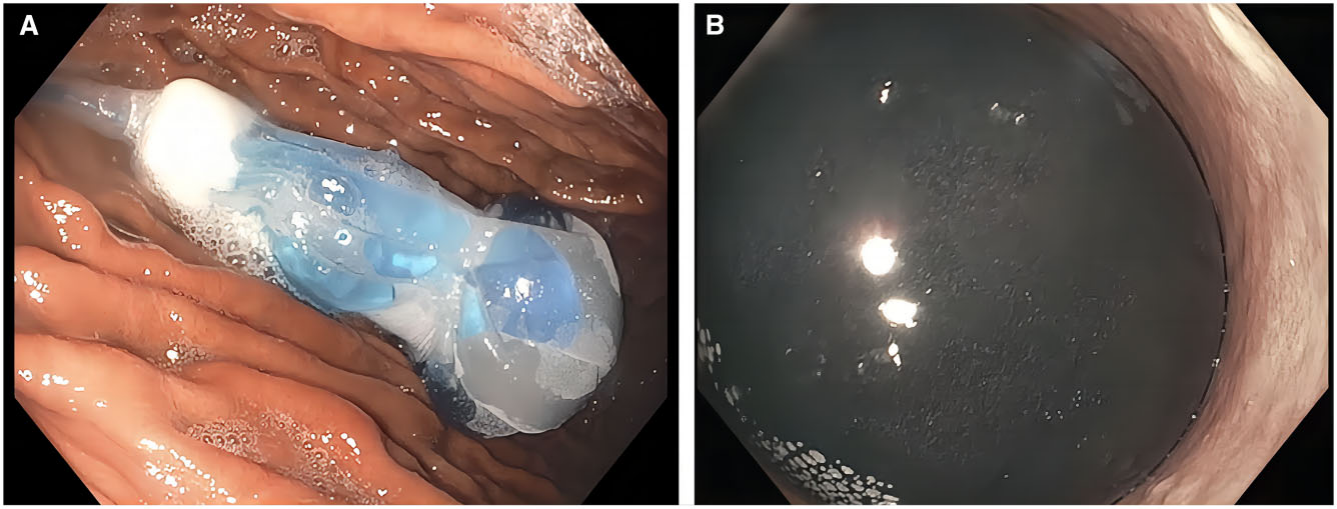
ORBERA™ Intragastric Balloon System (A) during and (B) after filling the balloon with fluid.

The OIBS is a single-chamber fluid-filled balloon that has been approved by the FDA for use in patients with a BMI of between 30 and 40 kg/m^2^ (Figure 1). It is used as both as primary therapy for weight loss and as a bridge to bariatric surgery. The volume that the balloon is typically filled with ranges from 400 to 700 mL depending on patient anatomy and the desired weight loss. This balloon is placed and removed endoscopically and remains in place for 6 months.

One post-regulatory study of the OIBS evaluating the safety and efficacy noted that of the final analysis of 321 patients at 6 months, total body weight losses of 5%, 10%, and 15% were 88%, 62%, and 31%, respectively, as well as a hemoglobin A1c (HbA1c) reduction of 0.7% at 6 months in pre-diabetic and diabetic patients [9]. Another pivotal multicenter randomized control trial evaluated 255 adults with BMI of 30–40 kg/m^2^, randomizing them into two arms: OIBS and lifestyle intervention or lifestyle intervention alone. At 6 months, patients in the lifestyle arm had a total body weight loss of 3.3% compared with 10.2% in the OIBS and lifestyle arm (*P *<* *0.0001). Higher total body weight loss and weight-loss maintenance were noted in the OIBS and lifestyle arm compared with the lifestyle arm alone at 9 and 12 months post balloon removal [10]. One study evaluated the efficacy of OIBS in the super obese (BMI > 40 kg/m^2^). This group noted significant reductions in median weight from 165.5 to 155 kg, BMI from 57.4 to 52.15 kg/m^2^, and excess weight loss of 12.9% at 6 months post balloon placement [11]. Sixty-three percent of the 46 patients in this study ultimately went on to undergo bariatric surgical interventions. Out of the 46 patients, 10 had complications, 7 of whom required early balloon removal. Results from this study, though encouraging, noted a slightly higher complication rate than studies evaluating the efficacy in OIBS in patients with a BMI of between 30 and 40 kg/m^2^.

Similarly, the ORBERA365™ system is a weight-loss aid approved for patients who have a BMI of between 27 and 50 kg/m^2^. The ORBERA365™ offers a longer 12-month period in which the balloon is placed. One prospective study evaluating the safety and efficacy in 97 patients who had the ORBERA365 balloon inserted and removed noted that the BMI dropped from 35.2 ± 4.4 to 29.8 ± 4.0 kg/m^2^ [12]. This balloon is not yet commercially available in the USA.

The Spatz3™ balloon is the first adjustable gastric balloon system to aid in weight loss for adult patients with obesity. The Spatz3™ balloon is initially filled to a volume of between 400 and 550 mL, depending on height and the presence of gastroesophageal reflux disease. The volume is typically increased by 250 mL if weight loss is not effective in the first 2 weeks or decreased by 100–150 mL if patients experience symptomatic intolerance. At 18 weeks, patients are then evaluated on whether they meet specific criteria (failure to reached goal weight and absence of gastroesophageal reflux disease or esophagitis, gastritis on endoscopy within this 18-week mark) and then can undergo further inflation of the balloon to a maximum of 1,000 mL with a goal of inducing greater weight loss. Its use is approved for ≤8 months in the USA, but it is not yet commercially available. One prospective multicenter randomized trial was designed with 231 patients (BMI of 30–40 kg/m^2^) that were randomized to receive a Spatz3™ adjustable IGB (aIGB) and lifestyle intervention vs lifestyle intervention alone. In the aIGB and lifestyle intervention group, patients achieved 15% (95% confidence interval, 13.9–16.1) total body weight loss at 32 weeks compared with 3.3% (95% confidence interval, 2.0–4.6) in the lifestyle intervention only group (*P *<* *0.0001) [13]. Though intolerance was common in ≤17% of patients, serious adverse events deemed to be device-related were observed in 4% of patients. There were no deaths.

The ReShape Duo balloon™, though not currently commercially available, is an endoscopically placed and removed fluid-filled balloon system with two chambers. Each chamber is filled with 450 mL of saline for a total of 900 mL. It is FDA-approved for 6 months of dwell time. In 2015, the REDUCE pivotal trial was a randomized control trial of the ReShape™ Duo balloon that randomized 326 patients (BMI of 30–40 kg/m^2^) to ReShape™ IGB in addition to a strictly monitored diet and exercise regime or sham endoscopy plus diet and exercise alone. At 24 weeks, patients who underwent the ReShape™ Duo balloon placement plus diet and exercise had significant excess weight loss of 25.1% intent-to-treat and 27.9% completed cases compared with the diet/exercise group alone (11.3% intention to treat and 12.3% completed cases) [14]. However, gastric ulceration was a common adverse event in this system, although this has been mitigated with device improvement.

The Obalon™ balloon, though not currently commercially available, is a gas-filled balloon system that is inflated with a nitrogen-sulfur hexafluoride gas mixture. A maximum of three balloons, each of which has a maximum inflation of 250 mL, can be deployed every 2–3 weeks for a total gastric occupying space of 750 mL. These balloons are ultimately removed endoscopically at 6 months. A small study in 2013 that evaluated patients who underwent balloon placement showed a mean weight loss at 6 months of 11 pounds [15]. The Six-Month Adjunctive Weight Reduction Trial in 2018 randomized 387 patients to 6 months of Obalon™ or sham capsule placement groups, followed by an additional 6 months of strictly monitored diet and exercise. At 5.5 months, the total body weight loss was 6.6% in the Obalon™ group and 3.4% in the sham group [16].

The Elipse™ balloon is not yet FDA-approved and is currently unavailable in the USA. This procedure-less balloon system is swallowed/ingested and naturally expelled by the gastrointestinal tract. This system entirely avoids the need for endoscopy. This balloon contains a volume of 550 mL and passage typically occurs 4 months after ingestion. A multicenter prospective analysis of 135 patients noted a mean total weight loss of 15.1% after 4 months. The most common symptom post-ingestion was nausea, with abdominal pain and diarrhea being common symptoms at the time of deflation [17]. One study pooled data across 19 international obesity centers and noted that among 1,770 consecutive patients who swallowed Elipse™ balloons, a 14.4% total body weight loss was achieved overall. A total of 99.9% of patients were able to safely swallow the balloon, 2.9% of patients developed intolerance necessitating balloon removal, and 0.6% of patients faced early balloons deflation; three small-bowel obstructions occurred necessitating laparoscopic surgery for removal. Statistically significant improvements in HbA1c were also noted from 5.1 ± 1.1 to 4.8 ± 0.8 at 4 months [18].

Outcomes vary across balloon type but treatment should be individualized based on patient presentation and desire. The aforementioned balloon systems are overall quite safe though there are a number of contraindications to balloon placement that are specific to each device. Contraindications generally include prior esophageal, gastric, or duodenal surgery or interventions, in addition to luminal or mucosal abnormalities including large hiatal hernias (>5 cm), esophageal or gastric varices, ulceration, severe coagulopathy, alcohol or drug addiction, and uncontrolled psychiatric illness.

### Other gastric devices

The Transpyloric shuttle™ (TPS; BAROnova, Inc., San Carlos, CA, USA) is FDA-cleared but not yet commercially available. The device is composed of a silicone catheter that has a flexible nature and connects to a smaller bulb. This bulb intermittently advances through the pylorus to intentionally induce a gastric outlet obstruction. It was originally FDA-approved in 2019 to be used in patients with a BMI of 30–40 kg/m^2^; it has a dwell time of 12 months. In the original feasibility study, 20 patients with a mean BMI of 36 kg/m^2^ were assigned to two groups to have the device for either 3 or 6 months. Patients with the TPS for 3 and 6 months had a mean total body weight loss of 8.9% and 14.5%, respectively [19]. A larger vital trial evaluating the TPS in 302 patients compared 270 TPS placements with 32 sham procedures. The total body weight loss at 12 months was 9.8% in patients who underwent TPS placement compared with 2.8% in the sham group. Twenty-nine patients developed adverse outcomes in this study, 10 of which were deemed serious. There were no reported deaths in this study [20].

Aspiration Therapy (AspireAssist^®^, Aspire Bariatrics, Inc.) was originally FDA-cleared in 2014 for patients with a BMI of between 35 and 55 kg/m^2^ The procedure involves placement of a A-tube, a percutaneously placed tube similar to a percutaneous endoscopic gastrostomy tube that connects directly to a gravity flow director system. Twenty to 30 minutes postprandially, the device can be activated by directly aspirating 30% of meal content that should then be wasted. Mechanistically, aspiration leads to the reduction of caloric absorption by the removal of luminal contents through the abdominal wall and has also been found to alter eating behaviors [21]. A randomized control trial comparing aspiration therapy with lifestyle and dietary counseling showed a mean excess weight loss of 31% at 12 months in the aspiration therapy group compared with 9.8% in the lifestyle change group [21]. A systematic review and meta-analysis that evaluated five studies across 590 patients noted that obesity-related co-morbidities including systolic and diastolic blood pressure, triglycerides, HbA1c, aspartate aminotransferase, and alanine aminotransferase all improved at 1 year following aspiration therapy. Weight loss at 1 year was also highly significant at 1 year following aspiration therapy [22]. In addition, 4-year outcomes regarding 58 patients who continued with the initial trial showed a mean total body weight loss of 14.2%, 15.3%, 16.6%, and 18.7% at 1, 2, 3, and 4 years, respectively [23].

## Gastric remodeling

### Endoscopic sleeve gastroplasty

**Figure 2.**
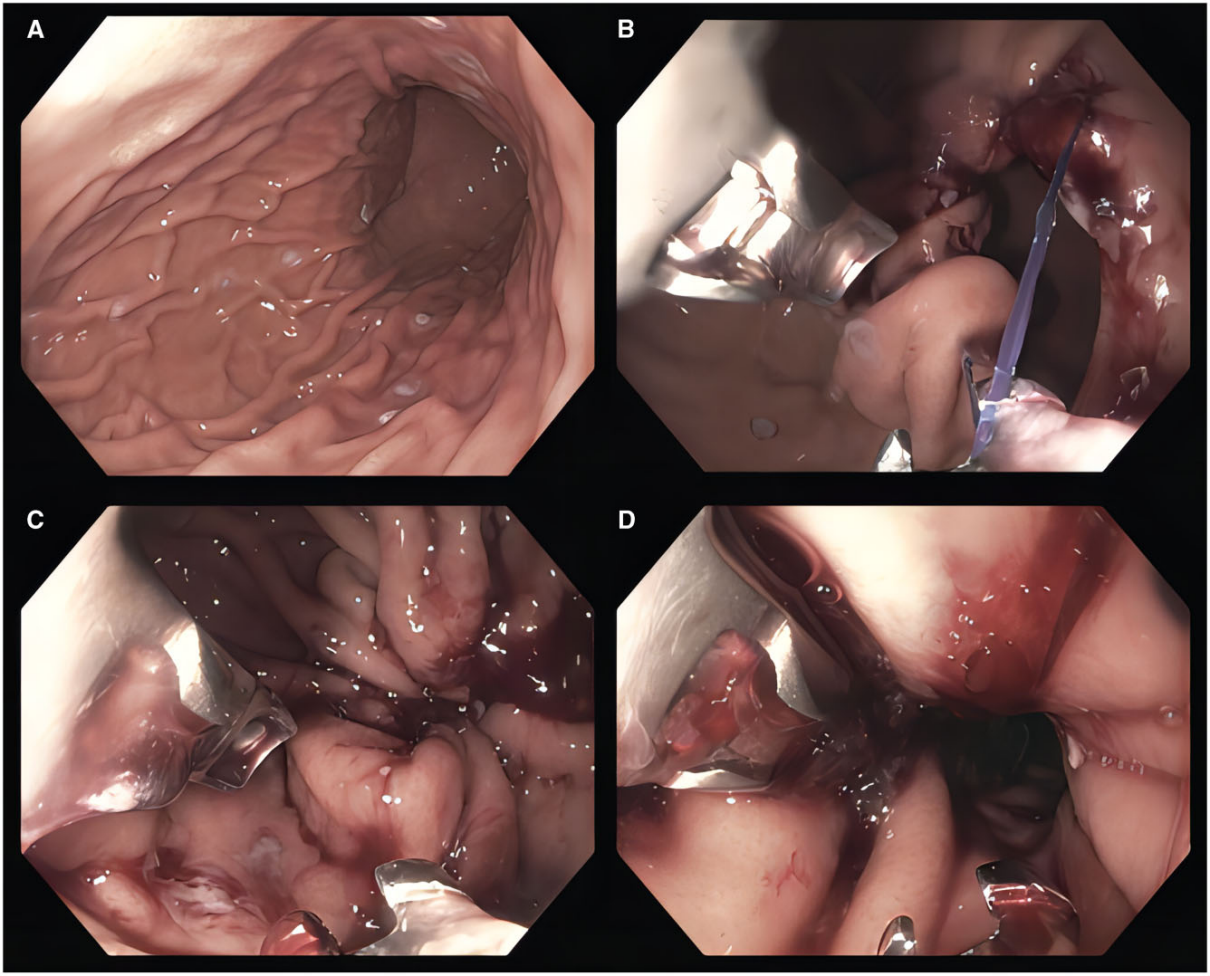
The procedure of endoscopic sleeve gastroplasty. (A) Marking of the anterior, greater curvature, and posterior surfaces with argon plasma coagulation prior to endoscopic suturing. (B) Following placement of the first suture. (C) Subsequent suture cinching, which is repeated numerous times moving proximally until the gastroesophageal junction is reached, leaving a small fundic pouch (D).

Endoscopic sleeve gastroplasty (ESG) is a gastric remodeling procedure that is FDA-approved for patients with a BMI of 30–50 kg/m^2^. This procedure is analogous to laparoscopic sleeve gastrectomy (LSG) but performed per-orally with an endoscopic suturing device that imbricates the entire greater curvature of the stomach (Figure 2) [24]. This procedure leads to reduction in gastric volume by 70%–80%, creating a narrowed luminal sleeve. Other hormonal changes occur including but not limited to changes in peptide tyrosine tyrosine and glucagon-like peptide secretion due to a decline in leptin and prevention of a rise in ghrelin secretion. These alterations are hypothesized to increase fullness, decrease hunger, and subsequently lead to greater weight loss [25, 26].

#### Technique

ESG is performed using an endoscopic suturing device (OverStitch, Apollo Endosurgery, Austin, TX, USA). The OverStitch Endoscopic suturing system allows placement of full-thickness sutures using a flexible endoscope. There are currently no published position statements or guidelines based on the consensus approach, but these are in development.

Generally, these procedures are performed under general anesthesia with endotracheal intubation. An esophageal overtube is typically placed to ensure safe atraumatic passage of the endoscope and suturing device [27]. Though practice patterns may vary, marking along the greater curvature, anterior, and posterior wall of the stomach with argon plasma coagulation may assist with suture placement. Sutures are commonly placed distally to proximally from the incisura to the gastroesophageal junction, leaving a small fundic pouch. Various suture patterns have been employed with the U-stitch (accordion approach) becoming the most popular technique [28]. Using this pattern, stitches are placed anteriorly, above the greater curvature, below the greater curvature, posteriorly, and then repeated creating a U-shape in the gastric lumen. This is then repeated a variable number of times from distal to proximal. This suture pattern narrows and foreshortens the gastric lumen. Suture pattern remains an area of interest and evolution with ongoing studies evaluating suture pattern as a predictor of long-term outcomes [29].

#### Efficacy and safety

Numerous retrospective and prospective studies have reported the efficacy and safety of ESG. This gastric remodeling procedure was first reported in 44 patients and demonstrated a mean weight loss of 16% at 6 months and 17.4% at 12 months [30]. A retrospective study analysing 248 patients who underwent ESG in three centers, two of which were in the USA, demonstrated 15.2% total body weight loss at 6 months and 18.5% total body weight loss at 24 months [31]. This study showed that the greatest predictor of 24-month weight loss was the percentage of total body weight loss at 6 months, demonstrating that failure to achieve substantial weight loss can be predicted early and adjunctive therapies should be offered to those patients. A prospective study of 216 patients that evaluated 5-year outcomes following ESG found that the mean total body weight loss was 15.9% with 60% of patients maintaining a total body weight loss of >10% [32]. There were no severe adverse events. A larger consecutive case series of 1,000 patients who underwent ESG followed for 18 months demonstrated 13.7%, 15%, and 14.8% at 6, 12, and 18 months of follow-up post-ESG, respectively [33]. Remarkably, 17 of 1,000 patients in this study had T2DM, 13 of whom were in complete remission of T2DM at 3-month follow-up post-ESG. This study also demonstrated the safety of ESG with a <1% risk of significant blood loss, severe abdominal pain or nausea, fevers, or the development of peri-gastric fluid collection. In a systematic review and meta-analysis of 1,772 patients who underwent ESG, the mean total body weight loss was noted to be 15.1% at 6 months and the sustained weight loss at 12 and 18–24 months was noted to be 16% and 17.2% [24]. The pooled rate of severe adverse events post-ESG was noted to be 2.2%. More recently, the first large-randomized clinical trial, the MERIT study, was performed across nine US centers, enrolling 209 participants to either the ESG with lifestyle modifications group (*n *=* *85) or lifestyle modifications alone (*n *=* *124) [34]. The primary outcome included the percentage of excess weight loss at 52 weeks. Secondary outcomes included the proportion of patients with ≥25% excess weight loss and percent total body weight loss. The mean excess weight loss at 52 weeks was 49.2% in the ESG group compared with 3.2% in the control group. The mean percent total body weight loss in the ESG group vs control group was 13.6% and 0.8%, respectively. At 52 weeks, the control group was able to crossover and undergo ESG and subsequently followed until 104 weeks. At 104 weeks, the primary ESG group had reached 83% excess weight loss and 41% total body weight loss. Participants in the crossover ESG group had an improvement in excess weight loss of 44.1%. At 104 weeks, the mean excess weight loss was 46.7% across all patients who underwent ESG (primary ESG and crossover ESG group) and 68% of participants in the primary ESG group maintained ≥25% of excess weight loss. In addition, 80% of participants in the ESG group had improvement in one or more metabolic co-morbidities at 52 weeks. In subjects with T2DM, HbA1c levels significantly improved in those participants who underwent ESG as compared with the control group, and 93% of those with T2DM in the ESG group had improvement in their T2DM compared with only 15% in the control group. In addition, ESG had marked benefit with regard to improvement in liver enzymes and the hepatic steatosis index. This study had a 2% severe adverse event rate, with no deaths, surgical interventions, or stay in an intensive care unit.

#### ESG vs LSG

Few studies have compared ESG with LSG. One study compared a cohort of 54 patients undergoing ESG with 83 patients undergoing LSG. At 6-month follow-up, total body weight loss was higher in the LSG group when compared with the ESG group (23.6% vs 17.1%), with caution that LSG had a higher rate of adverse events (16.9% vs 5.2%), a higher likelihood of new-onset gastroesophageal reflux disease post procedure (14.5% vs 1.9%), and a longer length of stay (0.34 vs 3.09 days) [35]. Similarly, another study evaluated patients with obesity (BMI > 30 kg/m^2^) who underwent ESG (*n *=* *91) and LSG (*n = *120) and measured total body weight loss at 3-month time intervals across 12 months. LSG achieved greater total body weight loss than ESG (29.28% vs 17.57%) but ESG had significantly less morbidity when compared with LSG, along with a significantly shorter length of stay [36]. A systematic review and meta-analysis comparing ESG with LSG at 12 months noted that the pooled total body weight loss of LSG was superior to that of ESG. Again, adverse events were significantly lower in participants undergoing ESG when compared with LSG [37].

#### Redo ESG

One major advantage to ESG is the ability to repeat the procedure in those with weight regain or plateau of weight loss. Several studies have supported the safety and efficacy of this procedure. In one study of 482 patients who underwent primary ESG, redo ESG was analysed in this patient cohort to evaluate its safety and efficacy [38]. In this retrospective analysis, 35 of 482 (7%) patients underwent a redo ESG, with a mean BMI of 33.6 kg/m^2^. There were groups including the weight-loss failure group (<10% total body weight loss at 6 months after primary ESG), the weight regain-lost group (≥10% total body weight loss and regained 50% of maximum weight loss at or after 1 year), and the weight plateau-lost group (≥10% total body weight loss but could not lose further over 3 months). In this study, total body weight loss was highest in the weight plateau-lost group (26%) compared with the weight-loss failure group (11%) and weight regain-lost group (12%). This study suggests that redo ESG may be most effective in patients with a plateau in their weight loss.

### Primary obesity surgery endoluminal

**Figure 3.**
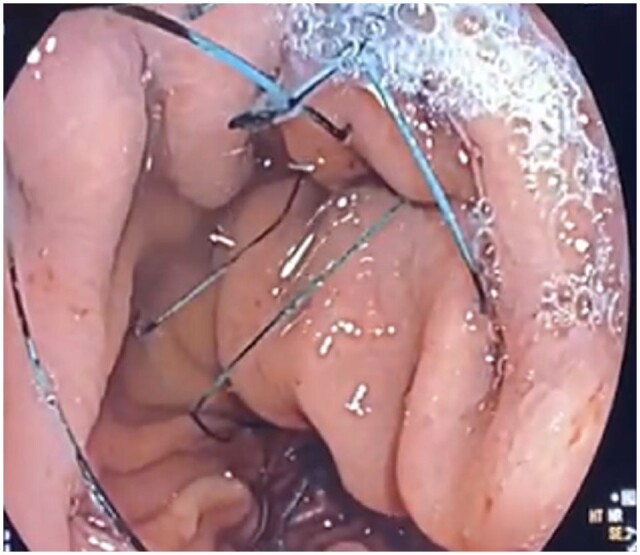
Plications placed during primary obesity surgery endoluminal 2.0.

Primary obesity surgery endoluminal (POSE) is an endoscopic plication technique that is performed using the Incisionless Operating Platform (USGI Medical, San Clemente, CA, USA). The Incisionless Operating Platform was initially FDA-approved in 2007 but is not currently widely available in the USA. POSE is performed with the creation of full-thickness gastric plications to reduce the volume of the gastric lumen [39, 40]. The procedure requires two operators and an overtube-style platform that has four working channels—one for the endoscope and three specialized instruments that help with plication and anchoring to the gastric wall [41]. A multicenter prospective randomized sham-controlled study (ESSENTIAL trial) compared the original POSE (*n *=* *221) with a sham treatment group (*n *=* *111) in patients with Class I and II obesity. At 1 year, the treatment arm had 4.95% total body weight loss compared with 1.38% in the sham treatment arm. This study did not meet the superiority margin between the two groups [42]. Insights from the ESSENTIAL trial have led to the development of technique adjustment including distal POSE and POSE 2.0 with significant improvement in outcomes, both of which focus more on the body and distal stomach than the fundus. Currently, the FDA has approved the expansion of POSE 2.0, a US pilot study studying POSE 2.0 in evaluating weight loss among patients with a BMI of 35–40 kg/m^2^ with obesity-related co-morbidities including hypertension or T2DM, across four centers in 35 subjects. This procedure places plications on the greater curvature of the stomach from the incisura to the proximal body, making the gastric reservoir for food much smaller (Figure 3) [43]. A recent prospective multicenter study evaluating POSE 2.0 across 44 patients yielded a mean percentage total body weight loss of 15.7% at 12 months, with improvements seen in hepatic steatosis as early as 6 months together with persistent improvements in hepatic steatosis for ≤24 months. Patients in this study reported increased fullness and reduced maximum meal volume with no serious adverse events occurring [44]. These procedures have been shown to be effective in weight loss and maintenance but also in improving other obesity-related co-morbidities, such as hepatic steatosis.

### Endomina gastric plication

**Figure 4.**
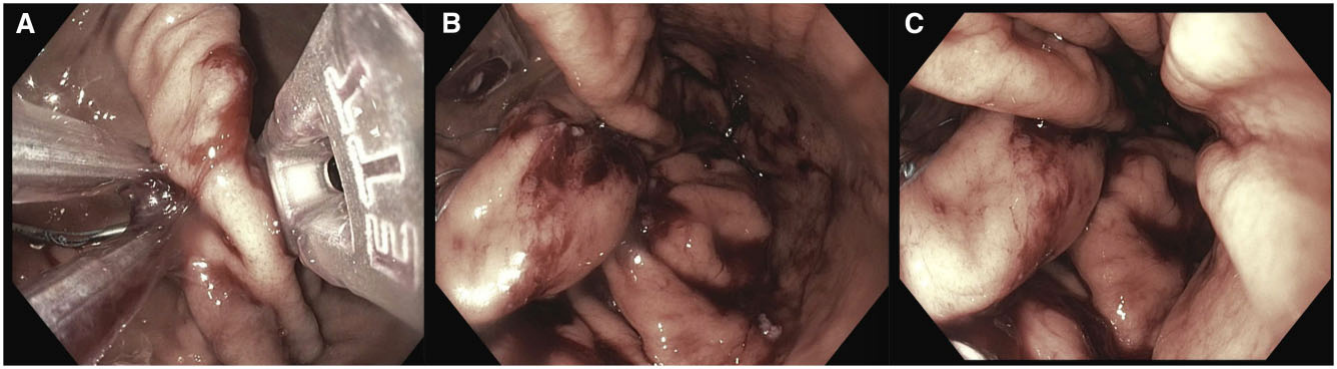
Endomina™ gastric remodeling procedure during the creation of plications (A) from distal to proximal (B) and (C).

The Endomina™ triangulation platform (Endo Tools, Goseelies, Belgium) is an alternative plication device that is assembled to a forward-viewing scope and used specifically for endoscopic gastric reduction. It accommodates a 5-Fr needle that passes a suture through full-thickness gastric folds (Transmural Antero-Posterior Endoscopic Suture). This device is advanced over two stiff guide wires into the gastric lumen. Once the Endomina™ system is advanced, it is opened and assembled with the endoscope fitting in between the aperture. The Transmural Antero-Posterior Endoscopic Suture is then advanced into the flexible arm of the platform and subsequently bent perpendicularly. Using forceps, the stomach wall is gripped and pulled through the channel of the endoscope in between the two arms of the platform (Figure 4). A needle is then advanced through two layers of the gastric wall and one pre-tied knot is released to create the first plication. The needle is subsequently retracted and the forceps opened to release gastric tissue. Once this is complete, a second plication is created on the opposite wall of the stomach. These steps are repeated from distal to proximal [45].

One study evaluated 51 patients across three centers in Europe who underwent ESG with use of the Endomina™ platform. A baseline BMI of 35.1 kg/m^2^ was noted and excess weight loss of 29% and total body weight loss of 7.4% was noted at 1-year follow-up. Of note, 88% of sutures were still in place across 30 patients who had follow-up gastroscopy at 1 year [45]. The same group performed a randomized controlled trial to evaluate short-term efficacy using Endomina™ for gastric remodeling, noting a 25% improved excess weight loss at 6 months compared with lifestyle modification alone [46]. A more recent study evaluating three different plication patterns in 48 patients undergoing Endomina™ found that the mean total body weight loss was 10.11% and excess weight loss was 42.56% with no differences noted between the three study groups [47]. Ongoing studies evaluating technique adjustment are currently being performed in the USA.

## Small-bowel devices

Endoscopic bariatric metabolic therapies focusing on small-bowel interventions are an evolving area of interest in endobariatrics. These therapies mimic some of the basic mechanisms of bariatric surgery, including small-bowel nutrient bypass and hormonal and insulin regulation. As a result, these interventions predominantly focus on promoting improved glycemic control (HbA1c) in patients with T2DM and improvements in liver-fat density.

### Duodenal sleeves

The EndoBarrier™ (GI Dynamics, Boston, MA, USA) sleeve is a gastrointestinal fluoropolymer liner that is 60 cm in length, impermeable, and is anchored in the duodenal bulb and extends to the proximal jejunum. These sleeves mimic the mechanism of duodenal exclusion seen in patients with Roux-en-Y gastric bypass (RYGB) anatomy. Mechanisms thought to help promote efficacy include changes in glucose metabolism, isolation of the duodenal mucosa from nutrient contact, isolation and delayed mixture of bile from nutrients, and a lack of expedited delivery of nutrients to the mid and hindgut similar to RYGB [48]. The duodenal sleeve is passed over a guide wire using endoscopic and fluoroscopic visualization. The device remains in place for 12 months with clinical trials showing improvements in HbA1c.

One multicenter study investigated EndoBarrier™ in 30 patients and compared weight-loss outcomes with 11 patients who had a low-calorie diet alone. The total body weight loss was 19.0% at 3 months in patients who received EndoBarrier™ compared with 6.9% for those on a low-calorie diet alone [49]. In this study, eight patients in the device group had T2DM, with significant decreases in HbA1c at 12 weeks of –1.1%; six of eight patients had decreased insulin dosages and/or oral diabetic medication after just 1 week of device placement. A systematic review and meta-analysis of 17 studies similarly found improvements in HbA1c at explant (–1.3%) and 6 months after explant (–0.9%) [50]. The original pivotal study in the USA was stopped early due to hepatic abscess formation. The protocol has since been modified and multicenter studies in the USA are currently underway.

Another endoluminal sleeve, the gastroduodenojejunal bypass sleeve (GJBS, ValenTx Endoluminal Bypass™; ValenTx Inc., Hopkins, MN, USA), is a 120-cm fluoropolymer sleeve that is delivered endoscopically under laparoscopic guidance. This was a historical alternative. The first trial evaluating the gastroduodenojejunal bypass sleeve liner in 24 patients with obesity found an average of 39.7% excess weight loss at 12 weeks. Significant improvements in HbA1c were also found [51]. Other pilot studies have shown similar findings [52].

### Incisionless anastomoses

The Incisionless Magnetic Anastomosis System™ (IMAS; GI Windows, West Bridgewater, MA, USA) is a magnet system capable of creating incisionless anastomoses between two points within the gastrointestinal tract. Porcine survival studies demonstrated successful creation of incisionless anastomoses that were fully patent and leak-free [53]. Human pilot studies subsequently evaluated the technically feasibility and clinical performance of the IMAS. Ten patients with obesity received the IMAS through delivery with a colonoscope under laparoscopic supervision. There were no device-related adverse events and the anastomosis remained patent over the 12-month study period. Total body weight loss was 14.6% in patients at 12 months and a significant reduction in HbA1c was noted in diabetic and pre-diabetic patients [54].

## Duodenal ablation devices

### Duodenal mucosal resurfacing

Revita™ duodenal mucosal resurfacing (DMR) uses circumferential hydrothermal energy to ablate the duodenal mucosa. A complete DMR typically consists of a zone of 9–10 cm. This ablation leads to regression of duodenal mucosa and improves insulin resistance [55]. Bypassing the duodenum enhances an incretin effect and subsequently increases insulin secretion. One study included 37 participants with T2DM who underwent complete DMR with substantial improvement in parameters of glycemia post-procedurally at 12 and 24 months. The mean HbA1c reduction at 12 months was 0.9% compared with baseline. The significance of this reduction is notable as the same degree of improvement as adding a second or third oral anti-diabetic medication [56, 57]. In addition, a consistent but important reduction in alanine aminotransferase was also observed from 40 ± 4 to 31 ± 2 U/L at 24 weeks, improving outcomes of non-alcoholic fatty liver disease as well. The Revita™ DMR is FDA-approved for investigational use. In trials with short-term follow-up, there have been significant improvements in HbA1c and net anti-hyperglycemic medication reductions [56, 58]. In a proof-of-concept study involving 39 patients with T2DM, HbA1c reduction of 1.2% was seen at 6 months across all participants [56]. In this study, patients had long duodenal segment ablation (average length: 9.3 cm) or short segment ablation (average length: 3.4 cm). HbA1c reduction was most notable in those patients who underwent long segment ablation (2.5% HbA1c reduction at 3 months and 1.8% at 6 months while on stable anti-diabetic medications). Furthermore, a pivotal double-blind randomized controlled trial (REVITA-2) evaluated the effect of DMR on glycemic control and liver-fat content using magnetic resonance imaging proton density liver-fat fraction in T2DM patients [59]. This 11-site multicenter trial randomized 109 patients 1:1 to DMR or a sham procedure. Improvements in HbA1c were –10.4 mmol/mol in DMR vs –7.1 mmol/mol in the sham group (*P *=* *0.147). Improvements in liver-fat change were also seen (–5.4% in DMR group vs –2.9% in the sham group, *P *=* *0.096). Despite not reaching significance, these improvements were seen consistently across groups and may potentially be meaningful in a larger sample. Lastly, a systematic review and meta-analysis in 2021 noted overall benefits in metabolic co-morbidities including hepatic steatosis and HbA1c across four studies [57]. Multicenter US pivotal studies are currently underway.

### Other ablative devices

There are currently a variety of novel ablative devices aimed at interfering with duodenal mucosa through mechanisms that do not involve heat. These are currently investigational.

## Weight regain following bariatric surgery

Weight recidivism after bariatric surgery is multifactorial. Although diet, lifestyle, and neurohormonal and psychological factors contribute, anatomic changes undoubtedly play a role. Technological advancements in the field of endobariatrics have led to many innovative approaches towards weight regain following bariatric surgery. These will be reviewed below for the two most common surgical procedures: RYGB and LSG.

### Weight regain following RYGB

#### Transoral outlet reduction

Weight recidivism after RYGB is common and commonly accompanied by a recurrence of obesity-related co-morbidities. Early data suggested that the size of the gastrojejunal anastomosis aperture was an independent predictor of and linearly correlated with weight regain. Surgical revision carries significant morbidity. As a result, less invasive options that can be performed endoscopically have emerged.

Transoral outlet reduction (TORe) is a procedure to reduce the diameter of an incompetent gastrojejunal anastomosis [60]. This has predominantly been performed with the use of endoscopic suturing, plication devices, and argon plasma coagulation. Other approaches such as cap-mounted clip placement, cryoballoon ablation [61], and radiofrequency ablation [62] have been reported in small series [63]. Sclerotherapy has also been reported but is no longer routinely performed [64, 65].

**Figure 5.**
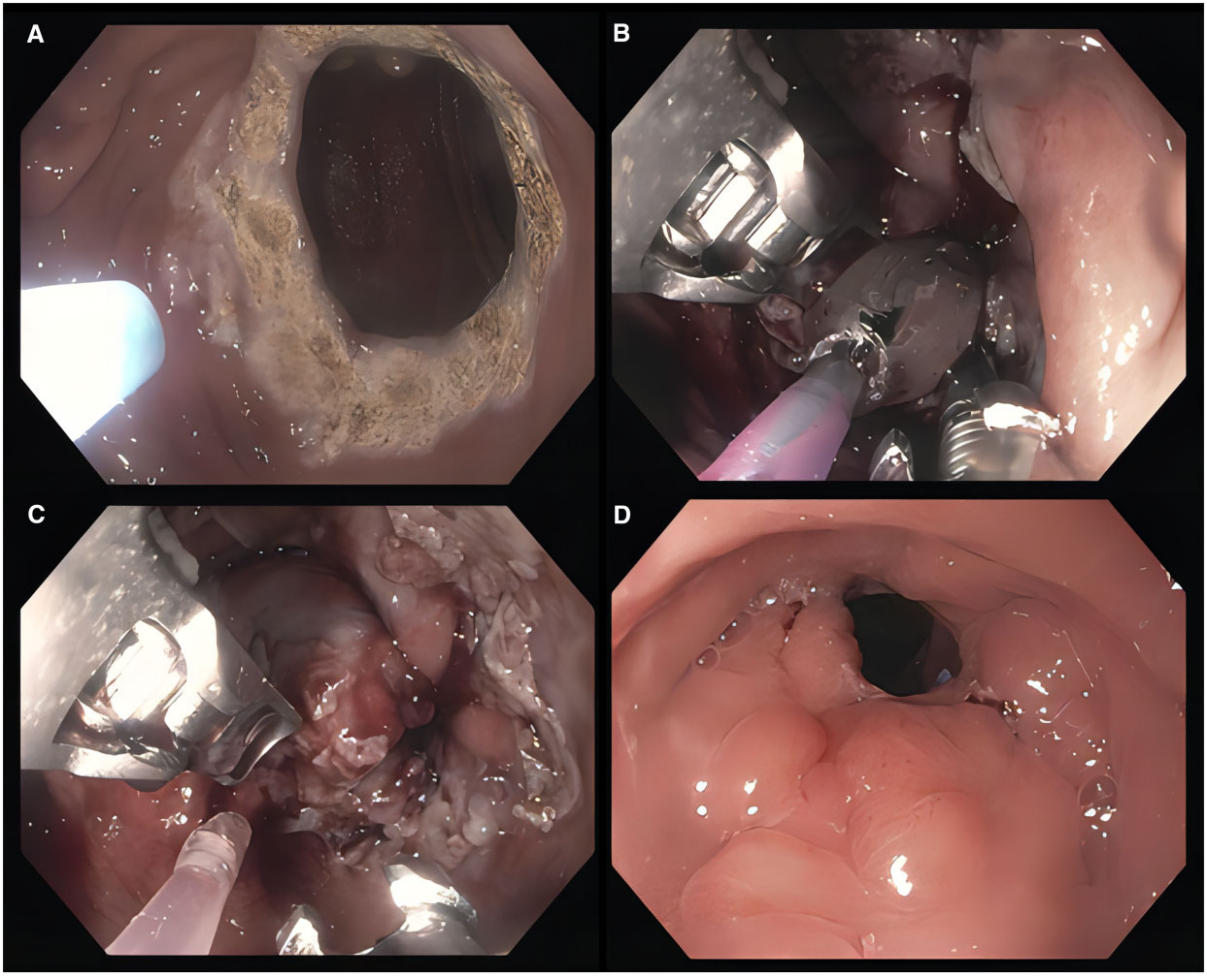
The procedure of transoral outlet reduction. (A) Marking of the dilated gastrojejunal anastomosis with argon plasma coagulation in preparation for purse-string suture placement. (B) The suture is cinched over a through-the-scope hydrostatic balloon and (C) ultimately removed demonstrating effective endoscopic revision. (D) Endoscopic evaluation 1 year later showed durability.

The most traditional approach to TORe includes argon plasma coagulation on the gastric side of the gastrojejunal anastomosis followed by endoscopic suturing using an endoscopic suturing device (OverStitch, Apollo Endosurgery, Austin, TX, USA) [66]. A variety of suture patterns including but not limited to interrupted and purse-string techniques have been described. An interrupted approach involves placement of individual sutures that are cinched sequentially. A purse-string pattern uses one suture to place multiple stitches in a continuous fashion circumferentially around the gastrojejunal anastomosis. After the completion of stitch placement, the suture is ultimately cinched over a through-the-scope hydrostatic balloon sized 6–10 mm (Figure 5). One study comparing interrupted and purse-string approaches found similar weight-loss results at 3 months but a significantly improved mean percent total body weight loss of 8.6% at 12 months in the purse-string group, suggesting more durability with the latter pattern [67]. The overall goal to reduce the anastomotic size may be more important than a decreased pouch volume, as suggested by several studies [68, 69]. Long-term data including 331 patients who underwent TORe using a purse-string approach found 8.8% total body weight loss at 5 years [70]. Newer iterations of this procedure including endoscopic submucosal dissection prior to endoscopic suturing and various pouch reduction techniques appear to enhance weight-loss outcomes. Argon plasma coagulation alone without concurrent endoscopic suturing is increasingly performed, reaching similar weight-loss outcomes in smaller gastrojejunal anastomosis diameters but commonly requiring numerous procedures [71].

Endoscopic plication devices have also been described for reduction in the diameter of the gastrojejunal anastomosis. The ROSE (Revisional Obesity Surgical Endoluminal) procedure uses the Incisionless Operating Platform. Similar to endoscopic suturing, this platform allows the reduction of the anastomotic diameter and pouch volume, and is commonly selected with longer pouch lengths. One prospective study of 20 patients demonstrated a 65% reduction in gastrojejunal anastomotic aperture size following ROSE, with total weight loss of 8.8 kg at 3 months [72]. Furthermore, a multicenter prospective series of 116 patients showed a 50% reduction in gastrojejunal anastomotic aperture size and a 44% reduction in pouch length [73]. Prospective trials are underway.

### Weight regain following LSG

#### Sleeve-in-sleeve

Weight regain after sleeve gastrectomy is common and increases dramatically at 5 years following the original surgery [74, 75]. Historically, patients with weight recidivism after sleeve gastrectomy were converted to RYGB at the discretion of the surgical team. Given the increasing number of patients undergoing LSG, the need for a less invasive endoscopic approach has surfaced. Endoscopic revision of a prior sleeve gastrectomy has been termed sleeve-in-sleeve. This procedure involves suturing or plication from distal to proximal. One multicenter study from 2020 evaluated 34 patients who underwent ESG after sleeve gastrectomy and noted a total body weight loss of 14.2%, 19.3%, and 17.5% at 1 year for patients with obesity Class I (BMI of 30–34 kg/m^2^), Class II (BMI of 35–39 kg/m^2^), and Class III (BMI ≥ 40 kg/m^2^), respectively, at 1 year [76].

## Conclusions

Endoscopic bariatric and metabolic therapy is a rapidly growing and exciting field, offering less invasive approaches to treat obesity and obesity-related co-morbidities. These procedures provide an alternative option for patients who do not desire or are not candidates for bariatric surgery. Numerous gastric devices and techniques are FDA-approved and commercially available, and many small-bowel therapies are under investigation. Future studies evaluating gastric and duodenal combination therapy, adjunctive pharmacotherapy, as well as individualized precision-health algorithms are underway.

## Authors’ Contributions

A.S. was responsible for drafting of the original manuscript; A.R.S. was responsible for the drafting and critical revision of the manuscript for important intellectual content. Both authors have read and approved the final version of the manuscript.

## Funding

None.
